# Assessment of the Chinese Resident Health Literacy Scale in a population-based sample in South China

**DOI:** 10.1186/s12889-015-1958-0

**Published:** 2015-07-11

**Authors:** Minxue Shen, Ming Hu, Siyun Liu, Yan Chang, Zhenqiu Sun

**Affiliations:** Department of Epidemiology and Health Statistics, School of Public Health, Central South University, 110 Xiangya Road, Changsha, 410078 Hunan China; Clinical Epidemiology Program, Ottawa Hospital Research Institute, 501 Smyth Road, Ottawa, K1G 8L6 ON Canada

**Keywords:** Health literacy, Item response theory, Confirmatory factor analysis, Measurement invariance

## Abstract

**Background:**

A national health literacy scale was developed in China in 2012, though no studies have validated it. In this investigation, we assessed the reliability, construct validity, and measurement invariance of that scale.

**Methods:**

A population-based sample of 3731 participants in Hunan Province was used to validate the Chinese Resident Health Literacy Scale based on item response theory and classical test theory (including split-half coefficient, Cronbach’s alpha, and confirmatory factor analysis). Measurement invariance was examined by differential item functioning.

**Results:**

The overall Cronbach’s alpha of the scale was 0.95 and Spearman-Brown coefficient 0.94. Confirmatory factor analysis showed that the test measured a unidimensional construct with three highly correlated factors. Highest discrimination was found among participants with limited to moderate health literacy. In all, 64 items were selected from the original scale based on factor loading, Pearson’s correlation coefficient, and discrimination and difficulty parameters in item response theory. Measurement invariance was significant but slight. According to the two-level linear model, health literacy was associated with education level, occupation, and income.

**Conclusions:**

The 2012 national health literacy scale was validated, and 64 items were selected based on classical test theory and item response theory. The revised version of the scale has strong psychometric properties with minor measurement invariance.

## Background

The concept of health literacy was introduced in China in 2005 by the Chinese government through a manual entitled “Basic Knowledge and Skills of People’s Health Literacy” [1, 2]. That manual used the definition of health literacy of the World Health Organization: the cognitive and social skills which determine the motivation and ability of individuals to gain access to, understand and use information in ways which promote and maintain good health [3]. Under that definition, health literacy goes beyond the narrow concept of health education and individual behavior-oriented communication: it addresses the environmental, political, and social factors that determine health. The US Institute of Medicine defines health literacy in a similar way: health literacy is a set of skills that enable people to participate more fully in society instead of simply functional capabilities [4]. The ability to read and write is the foundation for health literacy, upon which a range of complementary skills can be built [5].

Based on the existing situation in China, health literacy was defined by its government as a set of capabilities in three domains in 2008: conceptual knowledge and attitudes; behavior and lifestyle; and health-related skills. The first nationwide survey on health literacy in China was conducted in 2008, and it focused on health knowledge [6]. The second national survey was conducted in 2012, with an emphasis on basic reading ability, arithmetic, and understanding medical information [7].

Internationally, the most commonly used measure of health literacy is the Rapid Estimate of Adult Literacy in Medicine and its shortened version; these assess an adult patient’s ability to read common medical terms and lay expressions for body parts and illnesses [8–10]. The Test of Functional Health Literacy in Adults and its shortened version are timed tests of reading comprehension of medical information [11, 12]. Other measures of health literacy in clinical settings include the following: the Medical Achievement Reading Test; the Newest Vital Sign; the Set of Brief Screening Questions; Functional, Communicative and Critical Health Literacy; the eHealth Literacy Scale; the Cancer Health Literacy Test; and the Diabetes Numeracy Test [13–20]. These measures focus on a single dimension of health literacy, rather than identifying its multidimensional nature [21].

By contrast, some measures have expanded the scope of medical care-related literacy; they include the following: the Health Activity Literacy Scale; the Demographic Assessment of Health Literacy; the 2003 National Assessment of Adult Literacy; the Adult Literacy and Life Skills Survey; and the Health Literacy Assessment Using Talking Touchscreen Technology [22–27]. These scales and questionnaires are more comprehensive because they involve different health-related competencies. However, they are considered proxy measures owing to the lack of an explicit definition of the concept of health literacy [28].

In China, health literacy in clinical settings has been measured using translated versions of scales used overseas as well as original Chinese scales among certain populations [29–38]. For example, health literacy among older adults has been measured using the Chinese version of the Rapid Estimate of Adult Literacy in Medicine [33]; the translated version of the Test of Functional Health Literacy in Adults was employed to measure health literacy among adolescents aged 12–16 years in Nanning; the eHealth Literacy Scale was translated and used on a sample of senior high school students [34]; the Chinese version of the Diabetes Numeracy Test was used in a cluster-randomized trial in patients with diabetes [35]; and a three-question measure of health literacy derived from a systematic review was applied among cataract patients [36, 39]. However, all these studies investigated cross-sectional health literacy without evaluating the instruments employed [40–42]. In the present study, we assessed the reliability and construct validity of the Chinese Resident Health Literacy Scale based on item response theory (IRT) and classical test theory using a population-based sample from Hunan Province in 2012. We also examined the association between health literacy and sociodemographic factors.

## Methods

### Participants

The participants were residents aged 15 to 69 years who had lived in the sampled regions for more than 6 of the previous 12 months. Such individuals as patients, students, military personnel, and prisoners resident in hospitals, school dormitories, nursing homes, military bases, and prisons were excluded from the survey.

**Fig. 1:**
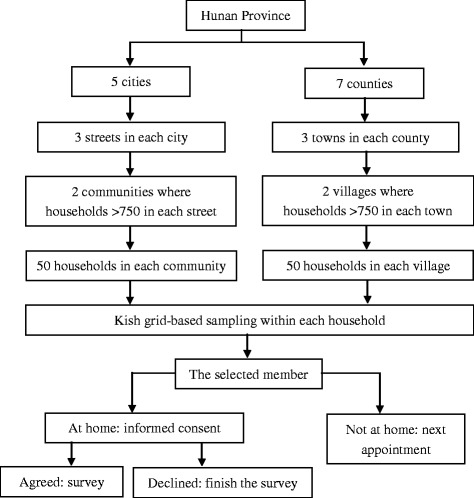
Study sampling process

We used a population-based stratified sampling frame, as shown in Fig. 1. The sampling strata included 13 cities or counties in Hunan Province, three streets or towns in each city or county, and two communities or villages (where the number of households exceeded 750) in each street or town. If there were fewer than 750 households within a community or village, the neighboring units were combined until that total was met. In each household, information regarding all family and non-relative members (e.g., hired nannies) aged 15–69 years who had been living there for more than 6 of the previous 12 months was recorded including gender (male or female) and age (elder to younger). One member in each house was selected for the survey by means of a Kish grid [43]. Unselected members were not allowed to complete the survey as a substitution.

The research protocol was reviewed and approved by the Medical Ethic Committee of the National Health and Family Planning Commission of China. All participants who agreed to participate in the study signed an informed consent form at the beginning of the survey.

### Study design

The Chinese Resident Health Literacy Scale was developed based on a manual published by the Chinese Ministry of Health in 2008—“Basic Knowledge and Skills of People’s Health Literacy” (trial edition) [1]. The scale was designed by experts in public health, health education and promotion, and clinical medicine using the Delphi method. Details of the development procedure have been described in a previous paper [44]. The scale contains 80 items and three dimensions: (1) knowledge and attitudes; (2) behavior and lifestyle; and (3) health-related skills. The questions cover six aspects: scientific views of health; infectious diseases; chronic diseases; safety and first aid; medical care; and health information. As indicated in Table 1, there are four types of questions in the scale: true-or-false; single-answer (only one correct answer in multiple-choice questions); multiple-answer (more than one correct answer in multiple-choice questions); and situation questions. With multiple-answer questions, a correct response had to contain all the correct answers and no wrong ones. Situation questions were given following a paragraph of instruction or medical information.

**Table 1:** Examples of items

Type of items	Examples	Dimension	Scope
True-or-false	• A01 - Antibiotic is effective in preventing influenza.	Knowledge and attitude	Infectious disease
	• A07 - Nutrients in vegetables and fruits are similar; so vegetables can be replaced by fruits.	Behavior and lifestyle	Chronic disease
Single-answer	• B01 - The integrated conception of health is: (1) Complete physical well-being without disease. (2) Physical and mental well-being. (3) A state of complete physical, mental, and social well-being and not merely the absence of disease or infirmity. (4) I do not know.	Knowledge and attitude	Scientific views of health
	• B36 - When the fire emergency occurs, the correct way to escape is: (1) Encase your head with your arms or cloths and rush out the fire. (2) Wet your cloths and head, or cover yourself with a wet towel and rush out the fire. (3) Flap the fire with you cloths and escape simultaneously. (4) I do not know.	Health-related skills	Safety and first aid
Multiple-answer	• C06 - Which of the following strategies can prevent chronic disease: (1) Quit smoking and limit the intake of alcohol. (2) Balance the nutrition. (3) Exercise moderately. (4) Be in good mood. (5) I do not know.	Knowledge and attitude	Chronic disease
	• C15 - Which descriptions about health management service for patients with type 2 diabetes are correct: (1) Only patients above 60 years old can receive the service. (2) All diagnosed patients in a community can receive the service. (3) Patients can receive four times of FBG testing for free. (4) Free FBG testing are unlimited, depending on the severity of disease. (5) I do not know.	Behavior and lifestyle	Medical care
Situation questions	• D03 - (A paragraph of instruction book for amoxicillin is given before the question) The drug may cause which of the following adverse reactions: (1) Nausea. (2) Depression. (3) Insomnia. (4) I do not know.	Health-related skills	Health information
	• D04 - (A paragraph of introduction to body mass index is given before the question) Mr. Li is 45 years old and 27.7 in BMI (kg/m^2^). Which of the following categories does he belong to, according to the Chinese adult BMI reference: (1) Obese. (2) Normal. (3) Overweight. (4) I do not know.	Health-related skills	Health information

Before the field study, a survey team was established in each of the 13 cities or counties; the team comprised a principal, a coordinator, four to six investigators, a quality controller, and a data manager. All these team members received training for the sampling method, research tools, and quality control. A simulated survey was conducted during the training, and the investigators’ eligibility was assessed before performing the field survey.

Written informed consent was obtained from all participants before the survey. The scale was self-administered. However, if a participant was unable to complete the scale owing to impaired vision or other such reasons, an interview was used as an alternative. In that situation, the investigators would complete the questions in a neutral fashion on behalf of the participants.

### Statistical analyses

Because repeated measures were not used, test-retest reliability was not determined. The split-half coefficient and Cronbach’s alpha were estimated before and after the item-selection procedure.

IRT was used to evaluate the precision of the measurements. IRT is a family of associated mathematical models that relate latent traits (ability) to the probability of responses to items in an assessment, and it has been widely used in psychometrics and health assessment [45]. It specifies a nonlinear relationship between binary, ordinal, or categorical responses and the latent trait (health literacy in this case). Compared with classical test theory approaches, the advantages of IRT include the following: near-equal interval measurement; representation of respondents and items on the same scale; and independence of person estimates from the particular set of items used for estimation [46].

We applied a two-parameter logistic IRT model for dichotomous responses. The two-parameter logistic model includes a difficulty parameter and discrimination parameter for each item. The difficulty parameter is the point on the ability scale that corresponds to a probability of a correct response of 0.5; the discrimination parameter estimates how well an item can differentiate among respondents with different levels of ability. Because the “I don’t know” choice was included for all questions, guessing parameters were not considered. Items with a discrimination parameter of 0.5 to 2.0 and a difficulty parameter corresponding to a certain region of the ability scale (−3.0 to 3.0) provide the most information [45, 47]. Parameters were estimated using a marginal maximum-likelihood method. The IRT model was recalibrated after the item-selection procedure. Measurement invariance of the scale among the different subgroups (by gender and race) was estimated using differential item functioning in the IRT model.

Pearson’s correlation coefficient was determined. An eligible items had to be significantly and at least moderately (0.4 to 0.7) correlated to the total score of its domain; hence, the correlation coefficient between them had to be above 0.4 [48]. The construct validity was assessed by confirmatory factor analysis (CFA). An assumed structure of the scale (three dimensions) was tested using a structural equation model. Since the items were binary measures, the unweighted least-squares method was employed for parameter estimation in the structural equation model. The chi-square value, goodness-of-fit index, root of the mean square residual, and parsimony goodness-of-fit index were used to assess the model fit. Several studies have recommended that the factor loading should be above 0.4 [49–51].

Items that met two or more of the following criteria were removed: (1) discrimination parameter <0.5 or >2.0; (2) difficulty parameter < −3.0 or >3.0; (3) factor loading <0.4; and (4) Pearson’s correlation coefficient <0.4. In addition, items with strong discrimination (≥1.0) were selected to form a short version of the scale.

The demographic variables were described, and raw scores among the different subgroups were compared using analysis of variance. After item selection, the association between health literacy scores and demographic variables was tested by means of a multilevel linear model.

The IRT calibrations were conducted using PARSCALE 4.1 (Scientific Software International Inc., Lincolnwood, USA). CFA was performed in AMOS 17.0 (Arbuckle JL and SPSS Inc., Chicago, USA). Multilevel model estimation was carried out with MLwiN 2.1 (Rasbash J, Charlton C, Browne WJ, Healy M, and Cameron B, Centre for Multilevel Modelling, University of Bristol, UK). Other analyses were conducted using SAS 9.2 (SAS Institute Inc., Cary, USA). The significance level was 0.05 for all statistical tests.

## Results

In all, 3900 participants were sampled, and 3731 (95.7 %) completed the survey without apparent logical errors or missing items. As indicated in Table 2, there were significant differences in the health literacy scores among the subgroups of age, education level, occupation, annual per capita income, and residence (*P* <0.05), but not among the subgroups of gender and race. The proportion of correct responses to the 80 items varied from 10.8 to 96.7 % (Table 2).

**Table 2:** Sociodemographic characteristics of the participants and association with health literacy scores

	N (%)	Health literacy scores	*P*-value
Gender			
Male	1890 (50.7)	50.9 ± 15.7	0.896
Female	1841 (49.3)	51.0 ± 16.0	
Age (year)			
15–24	345 (9.3)	54.7 ± 15.8	<0.001
25–34	592 (15.9)	54.3 ± 13.9	
35–44	901 (24.1)	52.0 ± 15.4	
45–54	846 (22.7)	49.9 ± 15.6	
55–64	781 (20.9)	47.5 ± 16.9	
65–69	266 (7.1)	48.7 ± 16.2	
Race			
Han	3445 (92.3)	50.9 ± 15.7	0.898
Minorities	276 (7.4)	50.8 ± 17.7	
Missing	10 (0.3)		
Education			
No formal education	225 (6.0)	40.5 ± 18.8	< 0.001
Primary school	1031 (27.6)	47.6 ± 16.8	
Junior school	1441 (38.6)	50.5 ± 15.1	
High school	691 (18.5)	55.4 ± 13.3	
College	319 (8.5)	60.8 ± 9.3	
Graduate school	11 (0.3)	61.0 ± 12.0	
Missing	13 (0.3)		
Occupation			
Civil servant	34 (0.9)	59.0 ± 12.0	< 0.001
Teacher	53 (1.4)	57.4 ± 10.2	
Medical staff	74 (2.0)	63.4 ± 10.8	
Student	68 (1.8)	55.1 ± 16.2	
Farmer	2610 (70.0)	48.7 ± 16.4	
Worker	188 (5.0)	53.1 ± 12.1	
Other	690 (18.5)	56.0 ± 12.9	
Missing	14 (0.4)		
Annual income per capita (CNY)			
≤ 10,000	1498 (40.2)	47.5 ± 15.7	< 0.001
10,001–20,000	883 (23.7)	50.7 ± 15.0	
20,001–30,000	73 (1.9)	57.9 ± 14.5	
> 30,000	857 (23.0)	55.5 ± 15.9	
Reject to answer	420 (11.2)		
Type of residence			
Urban	1458 (39.1)	52.0 ± 14.2	0.001
Rural	2273 (60.9)	50.3 ± 16.8	
Survey method			
Self-report	2870 (76.9)	51.1 ± 14.2	0.223
Interview	861 (23.1)	50.4 ± 20.4	

**Fig. 2:**
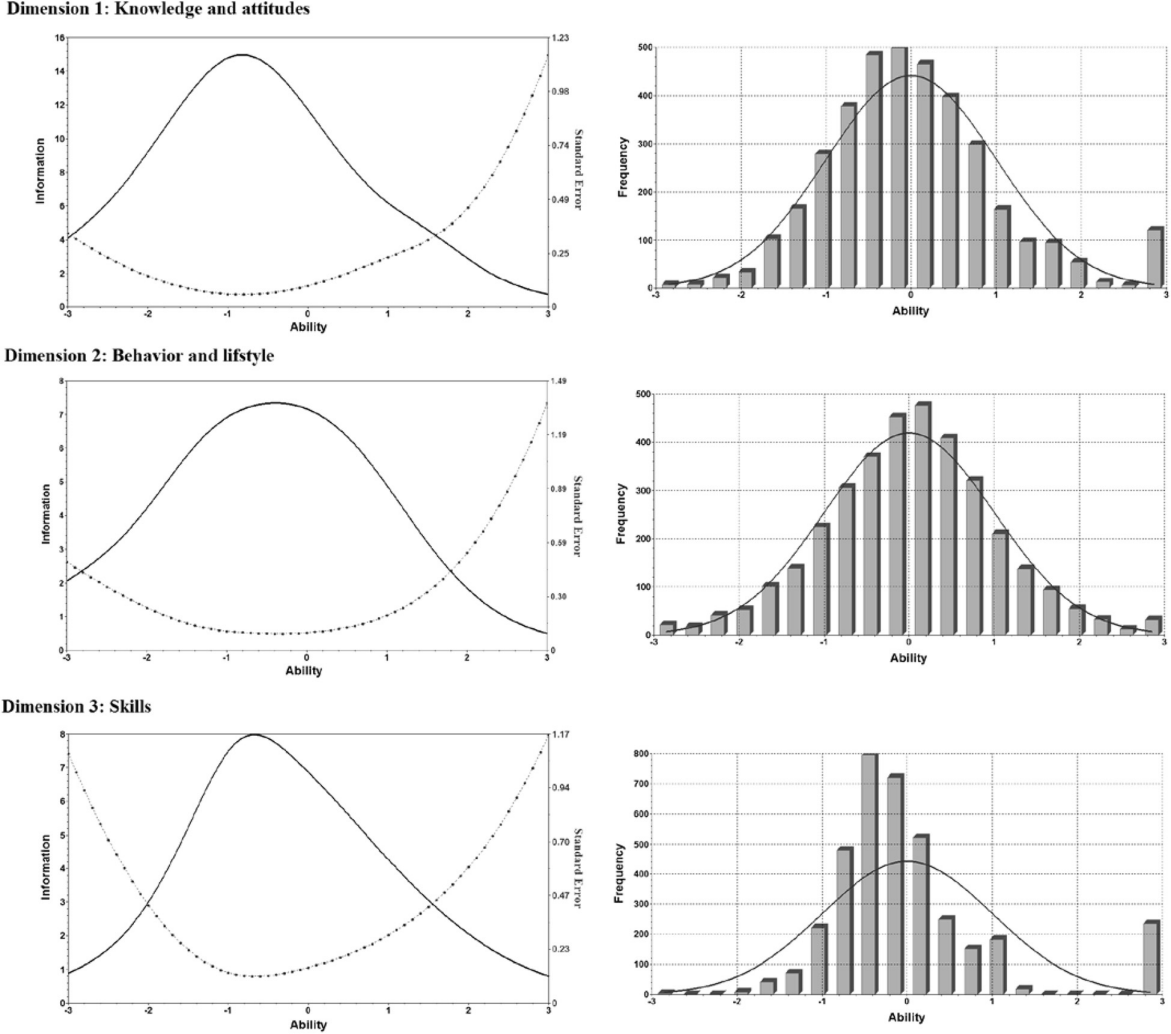
Test information and participant ability. Ability signifies health literacy estimated using the maximum-likelihood method. Ability in the item response theory (IRT) model practically (though not exclusively) ranged from −3 to +3. The test information reached a peak when the ability was between −1 and 0; this indicates that the measurement exhibited highest discriminative power among participants with limited and under-average ability with respect to health literacy

The Spearman–Brown split-half coefficient was 0.94. The overall Cronbach’s alpha was 0.95; Cronbach’s alpha of the three dimensions was as follows: 0.90 (knowledge and attitude, 38 items); 0.83 (behavior and lifestyle, 22 items); and 0.85 (skills, 20 items). The two-parameter logistic model fitted the data well (*P* >0.05). The difficulty and discrimination parameters from the IRT model appear in Table 3. Most items exhibited good discriminative power and moderate difficulty. As shown in Fig. 2, the test information reached a peak when the participants’ ability was between −1 and 0, which indicates that the measurement was most discriminative among participants with limited to medium-level abilities in health literacy.

**Table 3:** Evaluation of items based on item response theory and confirmatory factor analysis

		Correct (%)	Item correlation to dimension score	Factor loading	IRT parameters	
					Discrimination	Difficulty
Dimension 1: Knowledge and attitudes						
A01	Prevention of influenza	66.2	0.44	0.37	0.60	−0.82
A02	Hypertension	85.3	0.40	0.35	0.75	−1.78
A03	Health food	88.3	0.28	0.24	0.51	−2.67^a^
A04	Transfusion	64.4	0.51	0.47	0.80	−0.63
A05	AIDS detection and consultation	92.1	0.32	0.28	0.74	−2.43^a^
A06	Pregnancy and hazardous job	91.2	0.31	0.25	0.65	−2.54^a^
A08	Range of body temperature	76.5	0.27	0.20	0.33	−2.25^a^
A11	Food label	96.7	0.23	0.19	0.74	−3.21^a^
A15	Health examination	72.8	0.44	0.39	0.68	−1.11
B01	Definition of health	71.5	0.59	0.55	1.18	−0.79
B02	Improve resident’s health	78.4	0.56	0.50	1.19	−1.06
B03	Blood donation	80.4	0.50	0.46	0.99	−1.24
B04	Transmission of hepatitis B	70.7	0.51	0.48	0.83	−0.89
B05	Self-measured blood pressure	51.7	0.50	0.48	0.74	−0.11
B07	Early signals of cancer	60.4	0.59	0.57	1.06	−0.41
B08	Chronic diseases	56.7	0.50	0.49	0.76	−0.32
B09	Management of gas poisoning	80.4	0.49	0.43	0.93	−1.29
B10	Treatment of tuberculosis	80.9	0.40	0.36	0.66	−1.62
B11	Folic acid supplementation	56.5	0.53	0.53	0.85	−0.30
B12	Occupational protection	69.6	0.53	0.48	0.87	−0.81
B13	Iodine deficiency	71.9	0.53	0.51	0.95	−0.87
B14	Pesticide residues	41.0	0.31	0.31	0.40	0.56^a^
B18	Children immunization	85.4	0.47	0.42	0.99	−1.53
B23	Health knowledge	71.2	0.44	0.41	0.66	−1.04
B26	Warning icons	71.0	0.57	0.55	1.08	−0.80
B31	Adult pulse	72.7	0.49	0.46	0.81	−0.99
B34	Dog bite	90.4	0.29	0.21	0.57	−2.72^a^
B38	Expired food	87.0	0.46	0.42	1.01	−1.62
C02	Liver function	27.9	0.48	0.52	0.88	0.85
C03	Transmission of tuberculosis	30.4	0.43	0.47	0.72	0.83
C04	Child fever and rash	51.9	0.58	0.59	1.01	−0.12
C05	Osteoporosis	10.8	0.42	0.48	1.41	1.53
C06	Prevention of chronic diseases	43.4	0.55	0.55	0.91	0.20
C07	Health food selection	54.5	0.60	0.62	1.09	−0.21
C08	Dead livestock	70.5	0.52	0.50	0.86	−0.87
C17	Pesticide custody	61.7	0.34	0.29	0.32	−0.90^a^
D06	Weight control	70.9	0.50	0.47	0.73	−0.38
D07	Obesity-related disease	75.2	0.53	0.53	0.90	−0.86
Dimension 2: Behavior and lifestyle						
A07	Fruit and vegetable	75.2	0.40	0.36	0.53	−1.44
A09	Internet addiction	90.4	0.20	0.16	0.29	−4.74^a^
A10	Child and adolescent depression	87.6	0.37	0.32	0.68	−2.07^a^
A12	Lack of sleep	92.4	0.30	0.25	0.68	−2.60^a^
A13	Obtain health knowledge	93.3	0.30	0.26	0.72	−2.61^a^
A14	Attitude toward chronic diseases	57.0	0.38	0.32	0.41	−0.45^a^
B06	Smoking	54.9	0.55	0.63	0.83	−0.21
B15	Sport and electrolytes	70.0	0.48	0.44	0.71	−0.90
B16	Mental health	86.0	0.51	0.50	1.34	−1.34
B17	Public health service	46.3	0.56	0.52	0.88	0.12
B19	Fever symptom	79.3	0.42	0.33	0.64	−1.51
B20	Adverse drug reaction	79.0	0.39	0.31	0.57	−1.62^a^
B21	Health care card for pregnant women	52.6	0.45	0.40	0.57	−0.14
B27	Medical care procedure	71.8	0.57	0.54	1.14	−0.78
B29	House ventilation	65.7	0.50	0.45	0.79	−0.66
B39	Drug addiction	61.1	0.52	0.52	0.76	−0.48
C01	Mental health promotion	50.8	0.58	0.55	0.99	−0.05
C10	Bean products	23.4	0.52	0.49	1.03	1.00
C11	Sport and health	41.5	0.60	0.58	1.10	0.26
C13	Cough and sneeze	32.4	0.51	0.45	0.81	0.70
C14	Length of hospital stay	59.8	0.56	0.54	0.90	−0.39
C15	Management of type 2 diabetes	26.8	0.55	0.50	1.03	0.84
Dimension 3: Skills						
B22	Medical institutions	77.4	0.55	0.52	1.12	−0.88
B24	Infectious diseases	78.2	0.58	0.55	1.35	−0.82
B25	Food poisoning	78.6	0.57	0.54	1.36	−0.85
B28	OTC drugs	37.3	0.54	0.51	0.59	1.05
B30	Reading a thermometer	51.2	0.59	0.56	0.80	0.18
B32	Prenatal examination	41.2	0.44	0.39	0.38	1.05
B33	Condoms and STDs	84.2	0.52	0.51	1.19	−1.13
B35	Slight burns and scalds	70.9	0.49	0.44	0.69	−0.80
B36	Fire escape	77.8	0.46	0.41	0.66	−1.24
B37	Bleeding wound management	22.7	−0.04	−0.16	0.03	0.00^a^
B40	Electrical injury	70.6	0.43	0.40	0.42	−1.04
C09	Cardiopulmonary resuscitation	53.7	0.65	0.65	1.06	0.09
C12	Diabetes	43.8	0.51	0.46	0.53	0.60
C16	Brest feeding	42.3	0.51	0.50	0.56	0.70
C18	Lightning storms	76.9	0.44	0.40	0.58	−1.23
D01	Indications of amoxicillin	25.0	0.55	0.54	0.86	1.42
D02	Use of amoxicillin	64.4	0.50	0.43	0.55	−0.38
D03	Adverse reaction of amoxicillin	54.2	0.65	0.62	1.07	0.05
D04	Calculation of BMI	41.8	0.62	0.58	0.94	0.60
D05	Classification of BMI	45.7	0.59	0.53	0.77	0.46

^a^Items that met two or more criteria for deletion were removed

With the CFA results, the three-factor model showed slightly better fit than the one-factor model. Correlations among the three factors (knowledge and attitudes; behavior and lifestyle; skills) were 0.96–0.98, which indicates good evidence for unidimensionality, i.e., the dominant dimension of health literacy. Factor loading and the correlation coefficient between items and dimensional scores are presented in Table 3.

In all, 16 items were removed from the scale according to the criteria of item selection; 10 of them were true-or-false questions, which showed poor discriminative power and small factor loading. Sixty-four items were selected according to classical and modern test theory standards. The Spearman-Brown coefficient was 0.94. The overall Cronbach’s alpha was 0.95; Cronbach’s alpha of the three dimensions was as follows: 0.90 (knowledge and attitude, 30 items); 0.83 (behavior and lifestyle, 16 items); and 0.86 (skills, 18 items). Goodness-of-fit of the CFA and the IRT models improved slightly compared with the original scale. Factor loading, difficulty parameters, and discrimination parameters of all the items met the criteria.

A shorter version of the scale, comprising 19 items with discrimination parameters ≥1.0, was also created. The shorter version consisted of eight items in the knowledge and attitude dimension, five items in the behavior and lifestyle dimension, and six items in the health-related skill dimension. The overall Cronbach’s alpha was 0.88; Cronbach’s alpha of the three dimensions was 0.76, 0.64, and 0.77 respectively. The split-half coefficient was 0.87. The correlation coefficients and factor loadings of all the items were above 0.4 (mostly >0.5), and the discrimination parameters of all the items were 0.5–2.0 (mostly 1.0–2.0).

Differential item functioning in the IRT model was used to examine measurement invariance. The chi-square tests showed significant measurement invariance in both gender and race (*P* <0.05); however, the slope and threshold parameters were very close between male and female as well as between urban and rural groups.

The association between health literacy (revised scale) and demographic variables was explored using a two-level model because intracluster correlation was identified at the level of cities. As indicated in Table 4, education level, occupation, and income were associated with health literacy. Participants with higher socioeconomic status (higher education level and greater income) were more likely to have adequate health literacy. The intracluster correlation coefficient at the city level was 34.5 %.

**Table 4:** Association between health literacy and sociodemographic variables based on a two-level linear model

	Coefficient	95 % CI
Education		
No formal education	Reference	
Primary school	6.46	(4.74, 8.17)
Junior school	9.78	(8.06, 11.48)
High school	13.15	(11.22, 15.07)
College	18.34	(15.89, 20.78)
Graduate school	18.87	(11.50, 26.24)
Occupation		
Farmer	Reference	
Civil servant	4.41	(0.20, 8.61)
Teacher	2.02	(−1.50, 5.53)
Medical staff	7.76	(4.56, 10.95)
Student	−0.48	(−3.57, 2.61)
Worker	3.50	(1.64, 5.36)
Other	2.94	(1.59, 4.28)
Annual income per capita (CNY)		
< 10,000	Reference	
10,001–20,000	0.50	(−0.49, 1.48)
20,001–30,000	5.99	(3.28, 8.70)
> 30,000	0.70	(−0.41, 1.80)

## Discussion

To validate the scale used in the 2012 National Health Literacy Survey, we performed this study using a population-based sample in Hunan Province. Classical test theory (Cronbach’s alpha, split-half coefficient, and factor analysis) and modern test theory (IRT) were used in validating the scale. We found that the 2012 scale of health literacy meets psychometric standards. The overall Cronbach’s alpha was 0.95. The assumption that the scale measures a unidimensional construct was supported by the three-factor model fit being approximately that of the one-factor model fit and the three factors (knowledge and attitudes; behavior and lifestyle; skills) being highly correlated. Among the 80 items tested, 16 performed poorly and were removed. The remaining 64 items yielded a reliable estimate of health literacy, especially among participants with moderate and limited health literacy. The short version of the scale, which comprises 19 items with discrimination parameters ≥1.0, did not meet the standards for individual measurement (reliability ≥0.9). Nevertheless, the short version may still be effective for group comparisons [52].

In IRT, an item is useful only when it has good discrimination and its difficulty corresponds to a certain range in the ability scale: questions that are too hard or too easy provide little information [53]. However, if the discrimination is too high (i.e., greater than 2.5, as seen in clinical and psychological studies), the measured construct is often conceptually narrow. We limited the discrimination parameters to 0.5–2.0 because health literacy is a relatively broad concept. In this study, we identified items with inappropriate discrimination and difficulty. Most of them also had low factor loadings and correlation to the dimension score. However, the test used in the present study is time consuming. It usually took 30 min for an adult to complete the test; it took even longer for participants with limited literacy. Thus, in the future, it will be necessary to develop computerized adaptive testing and provide participants with short, tailored tests that have scores comparable to those of fixed-length tests.

Differential item functioning showed significant measurement invariance within both gender and race; however, the slope and threshold parameters were extremely close between the male and female as well as between the urban and rural groups. We observed no large differences between gender and race groups. The sample size in our study was sufficiently large to detect such slight differences. Thus, our results suggest that the Chinese Resident Health Literacy Scale may be efficiently applied for Chinese subjects of different genders and races for comparable scores.

The demographic factors associated with health literacy included education level, occupation, and annual income. Participants with higher education and better economic status were more likely to have adequate health literacy. Gender, age, race, and type of residence were found to be insignificant in the regression. The multilevel model identified an obvious intracluster correlation at the city level (primary unit in the sampling frame), with an intracluster correlation coefficient of 34.5 %. Health literacy is the outcome of health promotion, and both health literacy and socioeconomic factors are determinants of health. However, the potential of health education as a tool for promoting the social determinants of health has been neglected [54]. Health education should not focus only on changing personal lifestyles and improving compliance with disease management, but also on raising awareness of the social determinants of health [5].

Some limitations of this study deserve mention. First, we did not assess the content validity since the scale was initially developed by an expert panel from the Ministry of Health. Second, we did not perform repeated measures during the field study. Thus, the test-retest reliability was not determined. Third, as noted above, the test is time consuming: it usually took 30 min for an adult to complete and even longer for participants with limited literacy or other conditions.

Despite these limitations, this study has a number of implications. First, the original scale was found to be appropriate in terms of reliability and validity. We removed 16 items according to factor analysis and IRT, and the scores of the 64-item scale correlated highly with the scores of the original scale. Accordingly, the main conclusions of the 2012 National Health Literacy Survey were unaffected by validation of the scale it employed. Second, a shortened 19-item version was created because applying the original scale was very time consuming. The 19-item version was found to be slightly inferior to the original scale in terms of reliability (Cronbach’s alpha decreased from 0.95 to 0.88); however, it would still be effective for group comparisons and population studies. Third, the instruments used in the National Health Literacy Survey in 2008 and 2012 were different. Therefore, a direct comparison based on raw scores would be inappropriate. In the present study, IRT provided an opportunity for longitudinal comparison.

## Conclusions

We evaluated and revised the Chinese Resident Health Literacy Scale based on IRT and classical test theory using a population-based sample in Hunan, China. The revised 64-item scale was found to have strong psychometric properties and be free of obvious measurement invariance within the race and gender groups employed in this study. This is the first investigation to evaluate and revise the instrument used in the 2012 National Health Literacy Survey in China. The findings of this study support use of the new instrument in research into health literacy in public health settings, and this investigation offers useful implications for future studies.

## Abbreviations

IRT: Item response theory
CFA: Confirmatory factor analysis

## References

[1] Chinese Ministry of Health (2008). 66 tips of health: Chinese resident health literacy manual.

[2] Li XH (2008). Brief introduction on identification and dissemination of the Basic Knowledge and Skill of People’s Health Literacy by Chinese government. Chinese J Health Educ.

[3] World Health Organization (1998). Health promotion glossary.

[4] Institute of Medicine (2004). Health literacy: a prescription to end confusion.

[5] Nutbeam D (2008). The evolving concept of health literacy. Soc Sci Med.

[6] Wang P, Mao Q, Tao M, Tian X, Li Y, Qian L, Hu J, Ren X, Lv S, Cheng Y, Wei N, Yan L, Wei W, Du W, Xiao L, Xhou N (2010). Survey on the status of health literacy of Chinese residents in 2008. Chin J Health Educ.

[7] Li Y (2014). Introduction of 2012 Chinese residents health literacy monitoring program. Chin J Health Educ.

[8] Davis TC, Crouch MA, Long SW, Jackson RH, Bates P, George RB, Bairnsfather LE (1991). Rapid assessment of literacy levels of adult primary care patients. Fam Med.

[9] Bass PF, Wilson JF, Griffith CH (2003). A shortened instrument for literacy screening. J Gen Intern Med.

[10] Arozullah AM, Yarnold PR, Bennett CL, Soltysik RC, Wolf MS, Ferreira RM, Lee SY, Costello S, Shakir A, Denwood C, Bryant FB, Davis T (2007). Development and validation of a short-form, rapid estimate of adult literacy in medicine. Med Care.

[11] Parker RM, Baker DW, Williams MV, Nurss JR (1995). The test of functional health literacy in adults: a new instrument for measuring patients’ literacy skills. J Gen Intern Med.

[12] Baker DW, Williams MV, Parker RM, Gazmararian JA, Nurss J (1999). Development of a brief test to measure functional health literacy. Patient Educ Couns.

[13] Weiss BD, Mays MZ, Martz W, Castro KM, DeWalt DA, Pignone MP, Mockbee J, Hale FA (2005). Quick assessment of literacy in primary care: the newest vital sign. Ann Fam Med.

[14] Chew LD, Bradley KA, Boyko EJ (2004). Brief questions to identify patients with inadequate health literacy. Fam Med.

[15] Ishikawa H, Takeuchi T, Yano E (2008). Measuring functional, communicative, and critical health literacy among diabetic patients. Diabetes Care.

[16] Norman CD, Skinner HA (2006). eHEALS: The eHealth Literacy Scale. J Med Internet Res.

[17] Dumenci L, Matsuyama R, Riddle DL, Cartwright LA, Perera RA, Chung H, Siminoff LA (2014). Measurement of cancer health literacy and identification of patients with limited cancer health literacy. J Health Commun.

[18] Ishikawa H, Nomura K, Sato M, Yano E (2008). Developing a measure of communicative and critical health literacy: a pilot study of Japanese office workers. Health Promot Int.

[19] Huizinga MM, Elasy TA, Wallston KA, Cavanaugh K, Davis D, Gregory RP, Fuchs LS, Malone R, Cherrington A, Dewalt DA, Buse J, Pignone M, Rothman RL (2008). Development and validation of the Diabetes Numeracy Test (DNT). BMC Health Serv Res.

[20] Hanson-Divers EC (1997). Developing a medical achievement reading test to evaluate patient literacy skills: a preliminary study. J Health Care Poor Underserved.

[21] Malloy-Weir LJ, Charles C, Gafni A, Entwistle VA (2015). Empirical relationships between health literacy and treatment decision making: a scoping review of the literature. Patient Educ Couns.

[22] Hanchate AD, Ash AS, Gazmararian JA, Wolf MS, Paasche-Orlow MK (2008). The Demographic Assessment for Health Literacy (DAHL): a new tool for estimating associations between health literacy and outcomes in national surveys. J Gen Intern Med.

[23] Kutner M, Greenberg E, Jin Y, Paulsen C (2006). The health literacy of America’s adults: results from the 2003 National Assessment of Adult Literacy.

[24] Rudd RE (2007). Health literacy skills of U.S. adults. Am J Health Behav.

[25] Canadian Council on Learning (2007). Health literacy in Canada: initial results from the International Adult Literacy and Skills Survey 2007.

[26] Australian Bureau of Statistics (2008). Health literacy, Australia 2006.

[27] Hahn EA, Choi SW, Griffith JW, Yost KJ, Baker DW (2011). Health literacy assessment using talking touchscreen technology (Health LiTT): a new item response theory-based measure of health literacy. J Health Commun.

[28] Jordan JE, Osborne RH, Buchbinder R (2011). Critical appraisal of health literacy indices revealed variable underlying constructs, narrow content and psychometric weaknesses. J Clin Epidemiol.

[29] Leung AY, Cheung MK, Chi I (2015). Supplementing vitamin D through sunlight: associating health literacy with sunlight exposure behavior. Arch Gerontol Geriatr.

[30] Leung AY, Lou VW, Cheung MK, Chan SS, Chi I (2013). Development and validation of Chinese Health Literacy Scale for Diabetes. J Clin Nurs.

[31] Wang J, He Y, Jiang Q, Cai J, Wang W, Zeng Q, Miao J, Qi X, Chen J, Bian Q, Cai C, Ma N, Zhu Z, Zhang M (2013). Mental health literacy among residents in Shanghai. Shanghai Arch Psychiatry.

[32] Lam LT, Yang L (2014). Is low health literacy associated with overweight and obesity in adolescents: an epidemiology study in a 12-16 years old population, Nanning, China, 2012. Arch Public Health.

[33] Simon MA, Li Y, Dong X (2014). Levels of health literacy in a community-dwelling population of Chinese older adults. J Gerontol A Biol Sci Med Sci.

[34] Guo SJ, Yu XM, Sun YY, Nie D, Li XM, Wang L (2013). Adaptation and evaluation of Chinese version of eHEALS and its usage among senior high school students. Chin J Health Educ.

[35] Xu WH, Rothman RL, Li R, Chen Y, Xia Q, Fang H, Gao J, Yan Y, Zhou P, Jiang Y, Liu Y, Zhou F, Wang W, Chen M, Liu XY, Liu XN (2014). Improved self-management skills in Chinese diabetes patients through a comprehensive health literacy strategy: study protocol of a cluster randomized controlled trial. Trials.

[36] Lin X, Wang M, Zuo Y, Li M, Lin X, Zhu S, Zheng Y, Yu M, Lamoureux EL (2014). Health literacy, computer skills and quality of patient-physician communication in Chinese patients with cataract. PLoS One.

[37] Sun X, Yang S, Fisher EB, Shi Y, Wang Y, Zeng Q, Ji Y, Chang C, Du W (2014). Relationships of health literacy, health behavior, and health status regarding infectious respiratory diseases: application of a skill-based measure. J Health Commun.

[38] Sun X, Shi Y, Zeng Q, Wang Y, Du W, Wei N, Xie R, Chang C (2013). Determinants of health literacy and health behavior regarding infectious respiratory diseases: a pathway model. BMC Public Health.

[39] Powers BJ, Trinh JV, Bosworth HB (2010). Can this patient read and understand written health information. JAMA.

[40] Ye XH, Yang Y, Gao YH, Chen SD, Xu Y (2014). Status and determinants of health literacy among adolescents in Guangdong, China. Asian Pac J Cancer Prev.

[41] Wang C, Li H, Li L, Xu D, Kane RL, Meng Q (2013). Health literacy and ethnic disparities in health-related quality of life among rural women: results from a Chinese poor minority area. Health Qual Life Outcomes.

[42] Wang X, Guo H, Wang L, Li X, Huang M, Liu Z, Liu X, Wang K, Alamian A, Anderson JL (2015). Investigation of Residents’ Health Literacy Status and Its Risk Factors in Jiangsu Province of China. Asia Pac J Public Health.

[43] Kish L (1949). A procedure for objective respondent selection within the household. J Am Statist Assoc.

[44] Xiao L, Cheng YL, Ma Y, Chen GY, Hu JF, Li YH, Yang C, Tao MX (2008). A study on applying Delphi method for screening evaluation indexes of health literacy of China adults. Chin J Health Educ.

[45] Reise SP, Waller NG (2009). Item response theory and clinical measurement. Annu Rev Clin Psychol.

[46] Hambleton RK, Swaminathan H (1985). Item response theory: principles and applications.

[47] Baker FB (2001). The basics of item response theory.

[48] Richard T (1990). Interpretation of the correlation coefficient: a basic review. J Diagn Med Sonog.

[49] Gerbing DW, Anderson JC (1988). An updated paradigm for scale development incorporating unidemensionality and its assessment. J Marketing Res.

[50] Gorsuch RL (1997). Exploratory factor analysis: its role in item analysis. J Pers Assess.

[51] Velicer WF, Fava JL (1998). Effects of variable and subject sampling on factor pattern recovery. Psychol Methods.

[52] Nunnally JC, Bernstein IH (1994). Psychometric Theory.

[53] Thomas ML (2011). The value of item response theory in clinical assessment: a review. Assessment.

[54] Nutbeam D (2000). Health literacy as a public health goal: a challenge for contemporary health education and communication strategies into the 21st century. Health Promot Int.

